# Blood groups A and AB are associated with increased gastric cancer risk: evidence from a large genetic study and systematic review

**DOI:** 10.1186/s12885-019-5355-4

**Published:** 2019-02-21

**Authors:** Yingying Mao, Wenjun Yang, Qi Qi, Fei Yu, Tianpei Wang, Hongfei Zhang, Juncheng Dai, Hongxia Ma, Zhibin Hu, Hongbing Shen, Gang Li, Guangfu Jin

**Affiliations:** 1 Department of Epidemiology, Center for Global Health, School of Public Health, Nanjing Medical University, Nanjing, 211166 China; 2 Department of Epidemiology and Biostatistics, School of Public Health, Zhejiang Chinese Medical University, Hangzhou, 310053 China; 3 Key Laboratory of Fertility Preservation and Maintenance, The General Hospital, Ningxia Medical University, Yinchuan, 750003 Ningxia China; 4 Jiangsu Key Lab of Cancer Biomarkers, Prevention and Treatment, Collaborative Innovation Centre For Cancer Medicine, Nanjing Medical University, Nanjing, 211166 China; 5 Department of General Surgery, Jiangsu Cancer Hospital, Jiangsu Institute of Cancer Research, The Affiliated Cancer Hospital of Nanjing Medical University, Nanjing, 210009 China

**Keywords:** ABO blood group system, Gastric cancer, Case-control study, Systematic review, Meta-analysis

## Abstract

**Background:**

The association of ABO blood groups with gastric cancer risk was proposed decades ago, but the results have been inconsistent.

**Methods:**

We used two single nucleotide polymorphisms to determine ABO genotype in 4932 gastric cancer cases and 6158 controls of Chinese descent, and evaluated the associations of ABO blood groups and genotypes with risk of gastric cancer using multivariable logistic regression models. We also systematically reviewed published literature and performed a meta-analysis of all relevant studies.

**Results:**

In the case-control study, compared with blood group O, both blood group A and AB were associated with increased gastric cancer risk (for group A, odds ratio (OR) = 1.13, 95% confidence interval (CI): 1.02–1.24; for group AB, OR = 1.18, 95% CI: 1.02–1.36, respectively). Analyses of ABO genotypes revealed associations of *AO* and *AB* with risk of gastric cancer compared with *OO* genotype. Consistent with the case-control study, meta-analysis of 40 studies including 33,613 cases and 2,431,327 controls demonstrated that blood group A (OR = 1.19, 95% CI: 1.13–1.25) and AB (OR = 1.09, 95% CI: 1.03–1.16) were associated with increased risk of gastric cancer.

**Conclusions:**

Our analyses validated the association of blood group A with risk of gastric cancer, and suggested that blood group AB was also associated with gastric cancer risk. Functional investigations are warranted to elucidate the exact mechanism of ABO blood groups in gastric carcinogenesis.

**Electronic supplementary material:**

The online version of this article (10.1186/s12885-019-5355-4) contains supplementary material, which is available to authorized users.

## Background

Gastric cancer is the fifth most frequently diagnosed cancer and the third leading cause of cancer death worldwide, with estimated 1,033,701 new cases and 782,685 deaths in 2018 [1]. In China, gastric cancer is the second most common cancer type, with about 6,791,000 new cases in 2015 [2]. Common risk factors for gastric cancer includes *Helicobacter pylori* infection [3], smoking [4], drinking [5], and salted foods consumption [6]; however, these established risk factors could only explain a proportion of cases.

The ABO blood group system was discovered by Karl Landsteiner in 1900 [7], and it is by far the most important in human blood transfusions. The ABO blood type is controlled by a single gene, *ABO*, which encodes a glycosyltransferase that modifies the carbohydrate content of the red blood cell antigens. The role of ABO blood types in gastric cancer was initially suggested in more than 60 year ago, with the clinical observation that patients with gastric cancer were more likely to have blood group A than controls [8]. Since then, the association of ABO blood groups and gastric cancer risk has been extensively studied; however, the results have been variable. The inconsistent findings from these studies could possibly be attributed to small sample size which resulted in inadequate statistical power, poor study design that included inappropriate controls, and residual confounding from population heterogeneity.

Therefore, in the current study, we performed a large genetic study to evaluate the associations of ABO blood groups and genotypes with risk of gastric cancer in Chinese populations. In addition, we systematically reviewed published literature and conducted a meta-analysis of all relevant studies.

## Methods

### Study participants

Data were derived from three cohorts: Cohort I, the Nanjing/Beijing genome-wide association study (GWAS); Cohort II, the United States National Cancer Institute (NCI) GWAS, and Cohort III, a case-control study conducted in Jiangsu and Ningxia provinces. Among them, Cohort I and II have been described in detail previously [9, 10]. Briefly, for Cohort I, participants were from two separate studies conducted in Nanjing (565 cases and 1162 controls) and Beijing (468 cases and 1123 controls). Cases were patients with histopathologically confirmed gastric cancer, and controls were non-cancer individuals selected from local residents. For Cohort II, we obtained genotype data of gastric cancer cases and controls from the public database of Genotype and Phenotypes (dbGaP, https://www.ncbi.nlm.nih.gov/) (study accession number phs000361.v1.p1). Participants were from two separate studies conducted in Shanxi (1368 cases and 1650 controls from the Upper Gastrointestinal Cancer Genetics Project) and Linxian (257 cases and 450 controls from the Nutritional Intervention Trials). Cohort III was a case-control study conducted in Jiangsu (1615 cases and 1053 controls) and Ningxia (737 cases and 801 controls) provinces. Gastric cancer cases were collected from local hospitals and were histopathologically confirmed, and controls were cancer-free individuals selected from local residents.

All the study participants were unrelated individuals of Chinese descent. There was no overlap of participants between these studies. Written informed consent was obtained from all the study participants. The study protocols were approved by the relevant Institutional Review Boards.

### Assessment of ABO blood groups

For Cohorts I and II, information of genotyping, quality control and imputation has been described in detail elsewhere [9, 10]. ABO blood groups were determined using genetic data of two single nucleotide polymorphisms (SNPs) (rs8176746 and rs687289) in *ABO* to infer phased haplotypes for each participant with > 99% posterior probability [11]. Briefly, rs687289 is a proxy of rs8176719, which is a marker of the *O* allele. rs8176746 encodes exon 7 C796A, which is one of the seven standard ABO variants distinguishing *A* alleles from *B* alleles. Haplotype phase determination was required to distinguish *B* and *O* alleles from the more rare *A* and *O*-variant alleles [12]. As *A* and *O*-variant alleles represent a minority in Chinese populations, we assumed that these participants were of the *BO* blood group for the following analyses. For Cohort III, custom probes and primers were specifically designed for rs8176746 and rs687289 and genotyping was performed using TaqMan PCR-based assay (Applied Biosystems, Inc., Foster City, California), following the manufacturer’s instructions. The sequences of primers and minor groove binder (MGB) probes for rs8176746 were 5’-ACCGACCCCCCGAAGAA-3′ and 5’-CCAAGGACGAGGGCGATT-3′, and FAM-CCCCCAGGTAGTAGA and VIC-CCCCCATGTAGTAGAA. The primers and probes sequences for rs687289 were 5’-TCCCAGAACCAAGAGTGAAGTCA-3′ and 5’-CTGGGATATTGCTCACGTATGG-3′, and FAM-TGTTTCCAGGCCGTG and HEX-TGTTTCCAGACCGTGTC. The amplification reaction was done with 10 ng of template DNA, 2 × Hot Taq PCR reaction mix (Stegene BioTechnologies), primers and probes mix in 384-well plates using 7900 Fast Real-time PCR system (Applied Biosystems). Thermal cycling was performed under the following conditions: 50 °C for 2 min, 90 °C for 10 min, followed by 40 cycles of 95 °C for 15 s and 60 °C for 1 min. The call rates of rs8176746 and rs687289 were > 99%. Duplicate samples from 45 study participants were interspersed throughout the genotyping assays, and the concordance rate for these quality control samples were 100%.

### Systematic review and meta-analysis

We performed a systematic literature search in the PubMed database to identify all potentially relevant articles published from database inception through June 30, 2016. The search strategy included the terms “ABO” AND (“cancer” OR “carcinoma” OR “adenocarcinoma” OR “neoplasm”) AND (“gastric” OR “stomach”) in any text field of the database, without language limitations. The search identified 324 distinct publications. Two reviewers examined the publications independently to include studies in which frequencies, odds ratios (OR), or relative risks (RR) of the ABO blood group were reported for gastric cancer cases and controls. The same two reviewers manually checked the bibliographies of all relevant publications to identify possible additional studies for inclusion. We included data from original studies, and excluded those from secondary analyses or meta-analyses. For the studies that reported adjusted OR or RR with 95% confidence intervals (CI), we used the fully adjusted risk estimates as published. For the studies with only ABO blood group frequency data, we calculated and used the unadjusted effect estimates. Two reviewers independently assessed the quality of each included study using the modified Downs and Black Quality Assessment form. The quality score ranges from 0 to 14, and a higher score indicates better study quality. The discrepancies were resolved by consensus and discussion.

### Statistical analyses

For the case-control study, individual level data was pooled from the three cohorts. The differences in the distributions of demographic characteristics between cases and controls were assessed using Student *t* test for continuous variables and Pearson’s *χ*^2^-test for categorical variables. Deviations from Hardy-Weinberg equilibrium (HWE) among controls were assessed using *χ*^2^-based test. The association of ABO blood groups with gastric cancer risk was evaluated using multivariable unconditional logistic regression models, adjusting for age, sex, and study site. We further evaluated the association between gastric cancer risk and ABO genotypes (*AA*, *AO*, *BB*, and *BO* versus *OO*). Subgroup analyses were performed based on age, sex, study site and tumor subsite.

For the meta-analysis, heterogeneity across different studies was assessed using Cochran’s Q test and *I*-squared statistics. The fixed-effects model was used when there was no significant heterogeneity; otherwise the random-effects model was applied to provide more conservative estimates. The forest plots of the associations between blood groups and gastric cancer risk were generated for group A, B, AB versus group O. Publication bias was assessed by Egger’s regression and Begg’s rank correlation. The Begg’s funnel plots were generated, of which asymmetry was equated with the existence of potential publication bias. Sensitivity analyses were performed to evaluate the influence of individual studies on the overall effects by omitting one study at each time. We also evaluated possible heterogeneity of associations according to study subgroups, including study population, sample size, source of controls, study quality score and prevalence of *Helicobacter pylori* infection, which was classified as low and high as discussed by Peleteiro et al. [13] and Mentis et al. [14].

All statistical analyses were performed using Plink version 1.07, R software version 3.3.0 and STATA 11.0. Two-sided *P* values less than 0.05 were considered statistically significant, unless otherwise noted.

## Results

### Genetic analysis of case-control study

The present study included a total of 4932 gastric cancer cases and 6158 controls. Selected characteristics of the study participants are shown in Additional file 1: Table S1. The minor allele frequencies (MAF) of rs8176746 were 0.215 in gastric cancer cases and 0.223 in controls, and the MAFs of rs687289 were 0.447 in cases and 0.440 in controls. No apparent deviations from HWE in controls were observed for rs8176746 (*P* = 0.316) and rs687289 (*P* = 0.410), and no statistically significant associations were observed for rs8176746 or rs687289 with gastric cancer risk under the additive, recessive, dominant or co-dominant models.

Tables 1 and 2 show the phenotype and genotype distributions of ABO blood groups in gastric cancer cases and controls. The percentages of ABO blood groups in the control population were 30.95, 29.57, 30.59 and 8.88% for group O, A, B and AB, and the *ABO* allele frequencies were 55.93% for O, 21.77% for A and 22.30% for B, which were consistent with previous publications of Chinese populations [15–17]. Compared with blood group O, individuals with group A and AB had an increased risk of gastric cancer (for group A, OR = 1.13, 95% CI: 1.02–1.24, *P* = 0.018; for group AB, OR = 1.18, 95% CI: 1.02–1.36, *P* = 0.024, respectively). Similar results were observed for ABO genotypes. As shown in Table 3, compared with individuals with *OO* genotype, the risk of gastric cancer was 1.14-fold (1.03–1.26) for those with *AO* genotype and 1.18-fold (1.02–1.36) for those with *AB* genotype. (*P* = 0.015 for *AO* genotype, and *P* = 0.024 for *AB* genotype, respectively).

**Table 1:** Distribution of ABO blood group genotypes in gastric cancer cases and controls

rs8176746 × rs687289	No. of cases	Genotype frequency in cases, %	No. of controls	Genotype frequency in controls, %	Phenotype A alleles	Phenotype B alleles	Phenotype AB alleles	Phenotype O alleles
*AA*×*AA*	224	4.54	315	5.12		*BB*		
*AA*×*AG*	8	0.16	3	0.05		*BO* ^a^		
*AA*×*GG*	1	0.02	0	0.00		*BO* ^b^		
*AC*×*AA*	496	10.06	547	8.88			*AB*	
*AC*×*AG*	1146	23.24	1563	25.38		*BO*		
*AC*×*GG*	14	0.28	3	0.05		*BO* ^b^		
*CC*×*AA*	255	5.17	313	5.08	*AA*			
*CC*×*AG*	1308	26.52	1508	24.49	*AO*			
*CC*×*GG*	1480	30.01	1906	30.95				*OO*

^a^The AG haplotype corresponds to the rare *O24*, *O40* or *O41* alleles

^b^The AC× AG genotype denotes either haplotypes AA and CG (the common BO alleles, respectively) or AG and CA, which haplotype is rare in the Han Chinese population. The compound heterozygous genotypes require haplotype phase determination to distinguish *B* and *O* alleles from the more rare *A* and *O*-variant alleles. Since *A* and *O*-variant alleles together represent a minority in Chinese populations, we assumed that these individuals were of the *BO* blood group for these analyses

**Table 2:** Associations between ABO genotypes and gastric cancer risk^a^

Blood group	No. of cases	Phenotype frequency in cases, %	No. of controls	Phenotype frequency in controls, %	OR	95% CI	*P*
O (alleles *OO*)	1480	30.01	1906	30.95	1.00		
A (alleles *AA*, *AO*)	1563	31.69	1821	29.57	1.13	1.02–1.24	0.018*
B (alleles *BB*, *BO*)	1393	28.24	1884	30.59	0.96	0.87–1.06	0.410
AB (alleles *AB*)	496	10.06	547	8.88	1.18	1.02–1.36	0.024*

^a^OR, 95% CI and *P*-value were derived from unconditional logistic regression models adjusted for age, sex and study site

*OR* odds ratio, *CI* confidence interval

* indicates statistically significant

**Table 3:** Odds ratios (OR) and 95% confidence interval (CI) of the association between *ABO* genotypes and gastric cancer risk^a^

	First allele		
Second allele	*O*	*A*	*B*
*O*			
No. of cases	1480	1308	1169
No. of controls	1906	1508	1569
Multivariable-adjusted OR (95% CI)	Reference	1.14 (1.03–1.26)*	0.96 (0.87–1.07)
*A*			
No. of cases	–	255	–
No. of controls		313	
Multivariable-adjusted OR (95% CI)		1.07 (0.89–1.29)	
*B*			
No. of cases	–	496	224
No. of controls		547	315
Multivariable-adjusted OR (95% CI)		1.18 (1.02–1.36)*	0.95 (0.78–1.15)

^a^OR, 95% CI and *P*-value were derived from unconditional logistic regression models adjusted for age, sex, and study site

*OR* odds ratio, *CI* confidence interval

* indicates statistically significant

Additional file 2 Table S2 shows the subgroup analysis based on age, sex, study site and tumor subsite. Group A and AB were associated with gastric cancer risk in the subgroups of female (for group A, OR = 1.29, 95% CI: 1.07–1.56, *P* = 0.007; for group AB, OR = 1.42, 95% CI: 1.09–1.85, *P* = 0.010), younger participants (for group A, OR = 1.26, 95% CI: 1.09–1.46, *P* = 0.002; for group AB, OR = 1.37, 95% CI: 1.11–1.69, *P* = 0.003), Cohort II (for group A, OR = 1.26, 95% CI: 1.06–1.50, *P* = 0.008; for group AB, OR = 1.29, 95% CI: 1.01–1.65, *P* = 0.040), and non-cardia cancer (for group A, OR = 1.28, 95% CI: 1.13–1.45, *P* = 1.16 × 10^− 4^; for group AB, OR = 1.29, 95% CI: 1.07–1.55, *P* = 0.006). In the analyses limited to cardia cancer, blood group B was associated with decreased gastric cancer risk (OR = 0.84, 95% CI: 0.72–0.98, *P* = 0.027). No apparent evidence of heterogeneity was found between sexes or across different study sites, whereas significant heterogeneity was observed between different age groups (for group A, *P* = 0.044, *I*^*2*^ = 75.2%; for group AB, *P* = 0.048, *I*^*2*^ = 74.4%) and tumor sites (for group A, *P* = 5.00 × 10^− 4^, *I*^*2*^ = 91.8%).

### Systematic review and meta-analysis

The flowchart of the literature search and study inclusion is presented in Additional file 3: Figure S1. A total of 39 studies were identified as eligible for inclusion, including 32 case-control studies [15–31], 3 nested case-control studies [32–34] and 4 cohort studies [35–38]. The detailed characteristics of the 39 studies as well the present study are summarized in Additional file 4: Table S3. In total, 33,613 cases and 2,431,327 controls were used in the final meta-analysis.

**Fig. 1:**
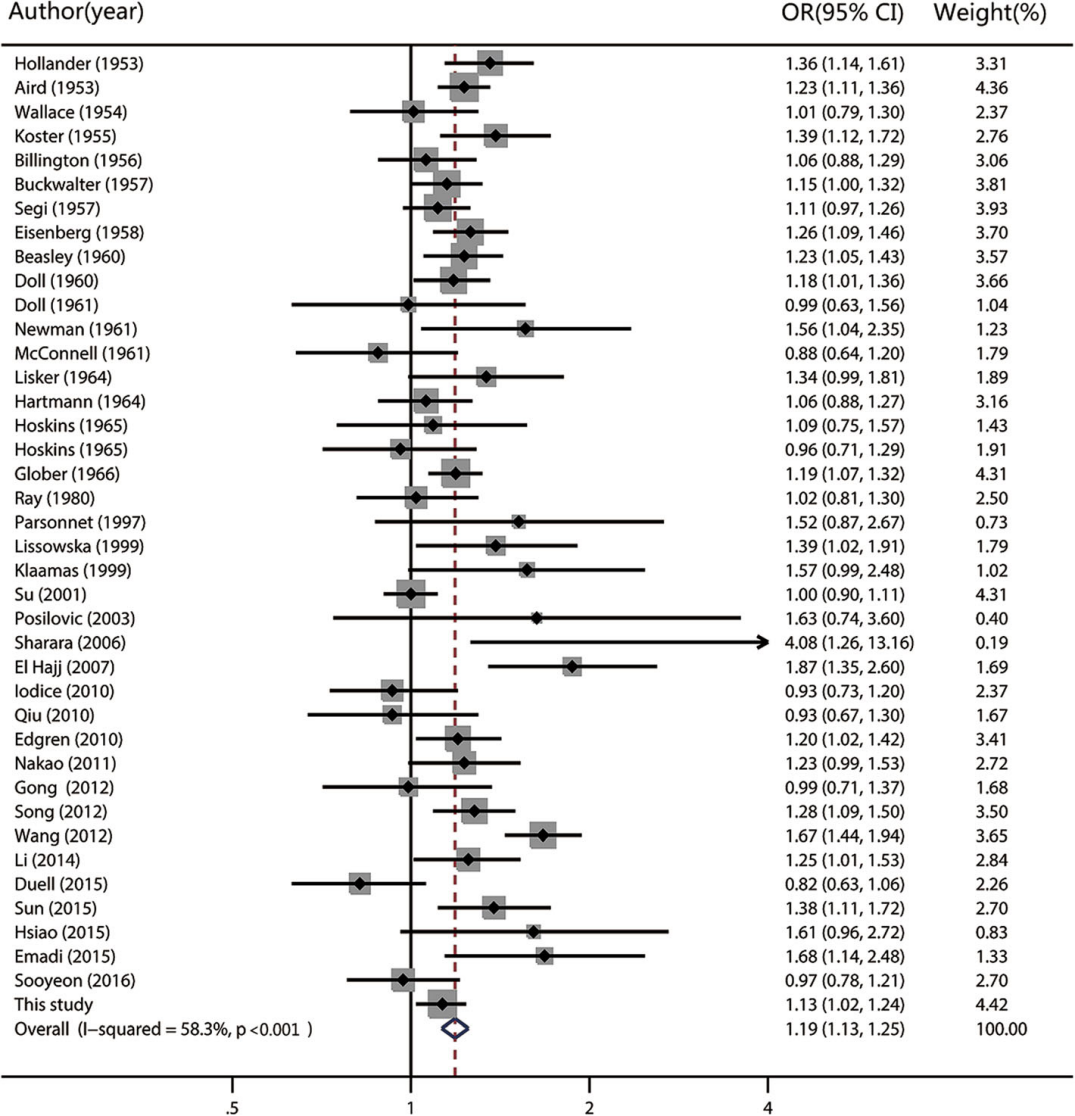
Random-effects meta-analysis forest plot of the odds ratio of gastric cancer according to blood group A with respect to group O. The studies are sorted by publication year. The solid squares are centered on the odds ratio (OR) point estimate from each study, and the horizontal line through each square indicates the 95% confidence interval (CI) for the study. The area of each square represents the magnitude of association, and the horizontal tips of the diamond represent the 95% CI

**Fig. 2:**
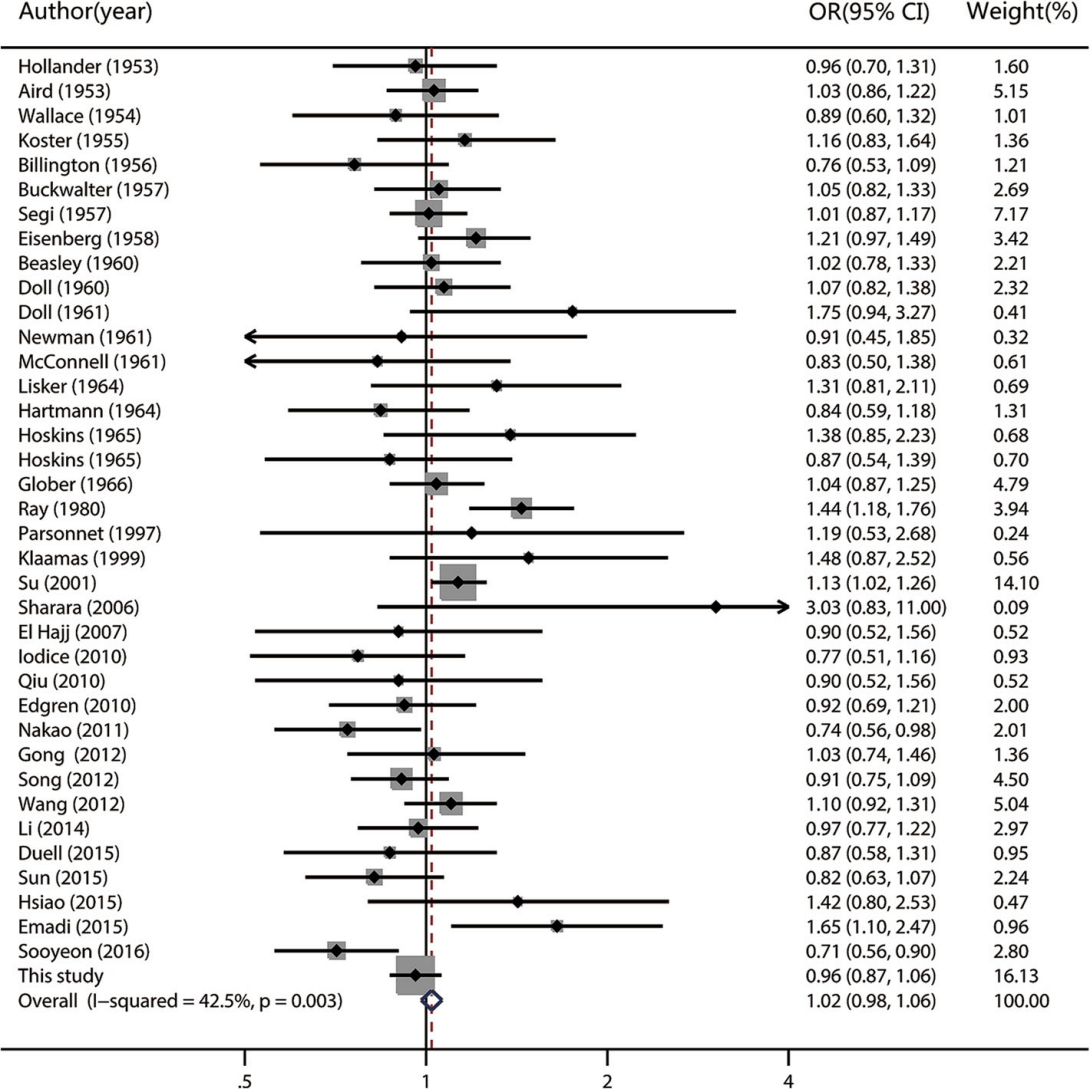
Fixed-effects meta-analysis forest plot of the odds ratio of gastric cancer according to blood group B with respect to group O. The studies are sorted by publication year. The solid squares are centered on the odds ratio (OR) point estimate from each study, and the horizontal line through each square indicates the 95% confidence interval (CI) for the study. The area of each square represents the magnitude of association, and the horizontal tips of the diamond represent the 95% CI

**Fig. 3:**
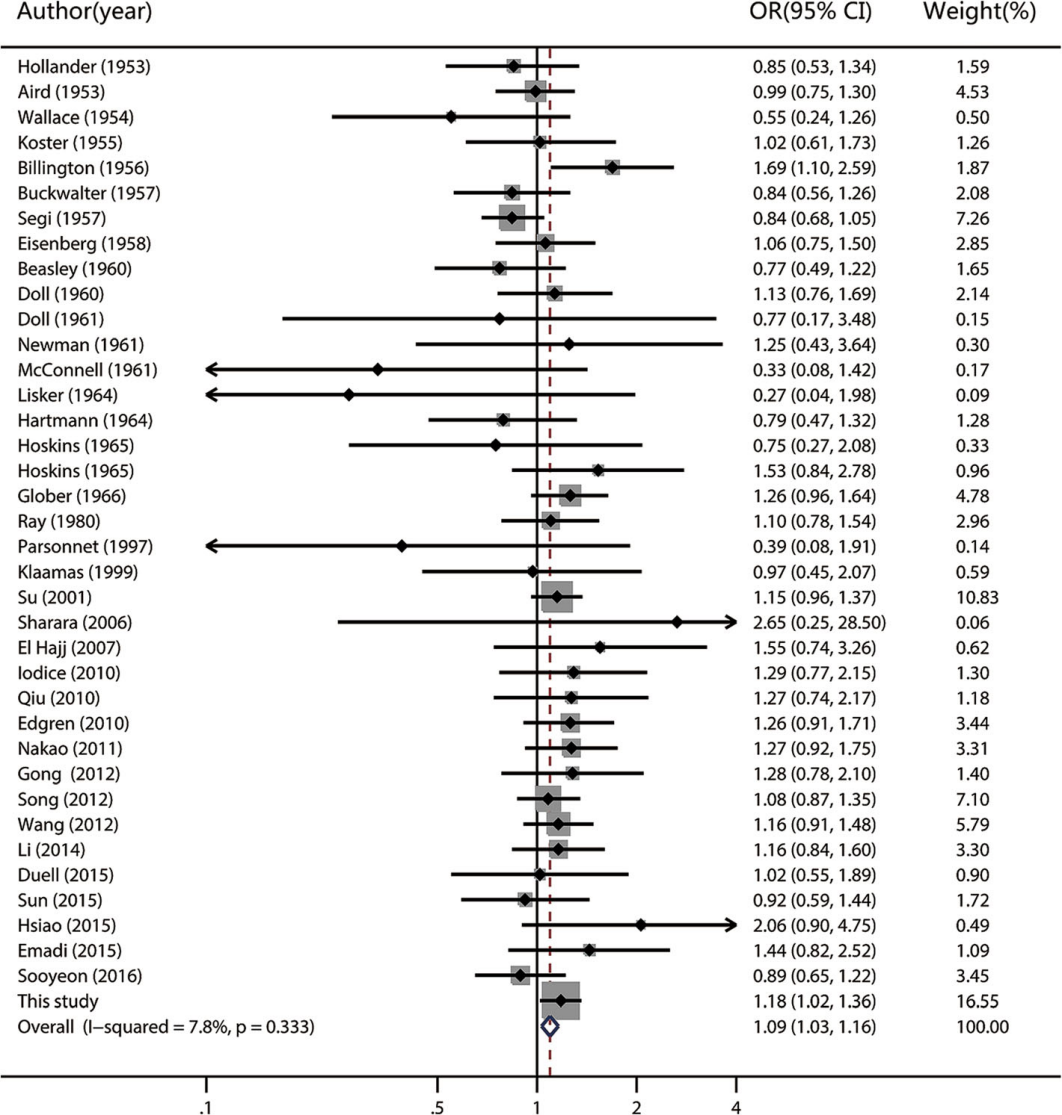
Fixed-effects meta-analysis forest plot of the odds ratio of gastric cancer according to blood group AB with respect to group O. The studies are sorted by publication year. The solid squares are centered on the odds ratio (OR) point estimate from each study, and the horizontal line through each square indicates the 95% confidence interval (CI) for the study. The area of each square represents the magnitude of association, and the horizontal tips of the diamond represent the 95% CI

Figures 1, 2 and 3 show the forest plots of the association of gastric cancer risk according to blood groups A, B and AB versus group O, respectively. Overall, compared with blood group O, group A and AB were associated with significant increased gastric cancer risk (for group A, OR = 1.19, 95% CI: 1.13–1.25; for group AB, OR = 1.09, 95% CI: 1.03–1.16, respectively). No statistically significant association was observed for group B (OR = 1.02, 95% CI: 0.98–1.06).

Sensitivity analyses showed that the pooled estimates did not change appreciably even if the most influential study was omitted. All Begg’s funnel plots appeared to be symmetrical (Additional file 5: Figure S2), and no significant asymmetry was found by the Egger’s and the Begg’s tests (*P* > 0.05).

Further stratification analyses are given in Additional file 6 Table S4. Blood group A was consistently associated with increased gastric cancer risk in different subgroups stratified by ethnicity, publication year, study sample size, source of control population, study quality score and prevalence of *Helicobacter pylori* infection. Group AB was associated with gastric cancer risk in the subgroups of Asian populations, studies published in the year 2000 and after, studies with higher quality score, larger sample size, including voluntary donors and studies conducted in areas with high prevalence of *Helicobacter pylori* infection. Group B was associated with increased gastric cancer risk in the subgroups of studies published before the year 2000, studies with larger sample size and studies including voluntary donors.

## Discussion

In the present study, using genetic data from large number of cases and controls, we found significant associations of blood group A and AB with increased risk of gastric cancer in Chinese populations. Consistent with the case-control study, meta-analysis including our study and 39 published studies confirmed that blood group A and AB were associated with gastric cancer risk.

The association of blood group A with gastric cancer has been observed in many previous studies, while only a few studies found significant association between blood group AB and gastric cancer risk. It has been hypothesized that the effect of group A on gastric cancer risk may be mediated by a small variety of physiological differences, which includes alterations in systemic inflammatory state, intercellular adhesion and membrane signaling, and immune surveillance for malignant cell. For example, Sievers et al. proposed that individuals with blood group A produced less free acid in their stomachs (the mean value of plasma pepsinogen was 494 units/ml vs. 564 units/ml) compared with those with group O [39]. Pare et al. reported that levels of soluble intercellular adhesion molecule 1 were significantly decreased for blood group subtype A101 versus O, but not for A201, B or AB versus O [40]. Notably, several recent studies suggested blood groups might be associated with altered inflammatory response to *Helicobacter pylori*, particularly cagA positive strains [24]. A case-control study and meta-analysis showed that gastric cancer patients from blood group A are more prone to be infected by *Helicobacter pylori* than individuals with other ABO blood types [19]. Ansari et al. also reported association between *Helicobacter pylori* BabA positive strain and blood group O non-Secretor [41]. In our study, stratification analysis by prevalence of *Helicobacter pylori* infection showed that blood group A was associated with gastric cancer risk in both subgroups with low and high prevalence, while group AB was associated with gastric cancer in the subgroup of high prevalence of *Helicobacter pylori* infection. We further analyzed the association of gastric cancer with the genetic variant rs10004195 in *TLR* locus (4p14) which was identified to be associated with *Helicobacter pylori* seroprevalence by GWAS [42] in a subset of our study participants. However, we did not observe statistically significant association between rs10004195 and gastric cancer risk, and no evidence of potential joint effect of ABO blood group and the genetic variant on gastric cancer risk was found (data not shown). Further studies are warranted to elucidate the relationship between ABO blood group, *Helicobacter pylori* infection and gastric carcinogenesis.

The strengths and potential limitations of this study deserve mention. In the case-control study, we used genotype-inferred blood groups which lowered the risk of misclassification from self-report blood type and allowed us to evaluate the associations of ABO genotypes with gastric cancer risk specifically. Because ABO blood type distribution varies considerably in different races, our study has another merit that we used an ethnically homogeneous population which mitigated ethnic differences in ABO distributions. Moreover, to the best of our knowledge, the current case-control study has the largest number of gastric cancer cases from multiple study centers. With regard to the meta-analysis, some points are worth considering. First, several studies included in the analysis involved controls from large groups of blood donors. Even through these volunteer donors are generally considered to be representative of their studies’ ethnic compositions, they may have other characteristics associated with altered risk of gastric cancer, such as younger age distribution and more prevalent in type O. However, analyses limiting studies not involving blood-donor controls did not change the effect estimates appreciably. Second, although we found significant heterogeneity among included studies for blood group A verses group O, we used random-effects models which allowed taking into account the heterogeneity among studies. Finally, because the prevalence of group *AA*, *BB* and *AB* was relatively low, thus, even though our meta-analysis involved large number of gastric cancer cases and controls, in some of the genotype categories subdivided by ethnicity, there were still insufficient numbers of participants to yield definitive conclusions.

## Conclusions

In conclusion, our analyses validated the association between blood group A and increased risk of gastric cancer, and indicated that group AB was also associated with gastric cancer risk. Further functional investigations are recommended to clarity the exact role of ABO in gastric carcinogenesis.

## Additional files

Additional file 1 **Table S1.** Selected characteristics of the study participants. (DOCX 17 kb)

Additional file 2 **Table S2.** Subgroup analyses of the associations between ABO blood types and gastric cancer risk. (DOCX 21 kb)

Additional file 3 **Figure S1.** The flowchart of literature search and study inclusion. (Docx 26 KB) (DOCX 26 kb)

Additional file 4 **Table S3.** Summaries of the studies included in the meta-analysis of ABO blood groups and gastric cancer risk. (DOCX 21 kb)

Additional file 5 **Figure S2.** Begg’s funnel plots for ABO blood group and gastric cancer risk. Figs. A-C are funnel plots for blood group A (A), B (B), AB (C) verse group O. The vertical axis represents the log-transformed odds ratios (ORs). The horizontal axis represents the standard errors (SEs) of log-transformed ORs. The funnel plots are drawn with 95% confidence intervals. (DOCX 80 kb)

Additional file 6 **Table S4.** Subgroup analyses stratified by potential modifying factors. (DOCX 20 kb)

## Abbreviations

CI: confidence interval
GC: gastric cancer
GWAS: genome-wide association studies
HWE: Hardy-Weinberg equilibrium
MAF: minor allele frequencies
NCI: National Cancer Institute
OR: odds ratio
RR: relative risk
SNP: single nucleotide polymorphism
