# Supplementary material for: Blood groups A and AB are associated with increased gastric cancer risk: evidence from a large genetic study and systematic review
Source: BMC Cancer. 2019 Feb 21;19:164. doi: 10.1186/s12885-019-5355-4 (PMC6385454; doi:10.1186/s12885-019-5355-4)
Supplement: Supplementary file 1 — Table S1. Selected characteristics of the study participants. (DOCX 17 kb) [file 12885_2019_5355_MOESM1_ESM.docx]

| Additional file 1: Table S1. Selected characteristics of the study participants. | | | | | | |  |  |  |  |  |  |
| --- | --- | --- | --- | --- | --- | --- | --- | --- | --- | --- | --- | --- |
|  | Nanjing/Beijing (Cohort I) | | |  | NCI (Cohort II) | |  | Jiangsu/Ningxia (Cohort III) | |  | Combined | |
|  | Cases | | Controls |  | Cases | Controls |  | Cases | Controls |  | Cases | Controls |
| Variables | n=1006 | | n=2273 |  | n=1625 | n=2100 |  | n=2301 | n=1785 |  | n=4932 | n=6158 |
| Age, N (%) | |  |  |  |  |  |  |  |  |  |  |  |
| <60 years | 548 (54.47) | | 1020 (44.87) |  | 731 (47.62) | 1000 (44.98) |  | 971 (42.20) | 691 (38.71) |  | 2250 (45.62) | 2711 (44.03) |
| ≥60 years | 458 (45.53) | | 1253 (55.13) |  | 894 (52.38) | 1100 (55.02) |  | 1330 (57.80) | 1094 (61.29) |  | 2682 (54.38) | 3447 (55.97) |
| Sex, N (%) |  | |  |  |  |  |  |  |  |  |  |  |
| Male | 714 (70.97) | | 1696 (74.62) |  | 1260 (77.54) | 1430 (68.10) |  | 1731 (75.23) | 1048 (58.71) |  | 3705 (75.12) | 4174 (67.78) |
| Female | 292 (29.03) | | 577 (25.38) |  | 365 (22.46) | 670 (31.90) |  | 570 (24.77) | 737 (41.29) |  | 1227 (24.88) | 1984 (32.22) |
