# Supplementary material for: Blood groups A and AB are associated with increased gastric cancer risk: evidence from a large genetic study and systematic review
Source: BMC Cancer. 2019 Feb 21;19:164. doi: 10.1186/s12885-019-5355-4 (PMC6385454; doi:10.1186/s12885-019-5355-4)
Supplement: Supplementary file 2 — Table S2. Subgroup analyses of the associations between ABO blood types and gastric cancer risk. (DOCX 21 kb) [file 12885_2019_5355_MOESM2_ESM.docx]

| Additional file 2: Table S2. Subgroup analyses of the associations between ABO blood types and gastric cancer risk. | | | | |
| --- | --- | --- | --- | --- |
|  | Type O | Type A | Type B | Type AB |
| Age |  |  |  |  |
| <60 years |  |  |  |  |
| Cases, N (%) | 638 (28.36) | 748 (33.33) | 619 (27.59) | 245 (10.92) |
| Controls, N (%) | 872 (32.17) | 803 (29.62) | 799 (29.47) | 237 (8.74) |
| OR (95% CI) | Reference | 1.26 (1.09-1.46) | 1.02 (0.88-1.19) | 1.37 (1.11-1.69) |
| *P* |  | 0.002 | 0.748 | 0.003 |
| ≥60 years |  |  |  |  |
| Cases, N (%) | 842 (31.39) | 815 (30.39) | 774 (28.86) | 251 (9.36) |
| Controls, N (%) | 1034 (30.00) | 1018 (29.53) | 1085 (31.48) | 310 (8.99) |
| OR (95% CI) | Reference | 1.03 (0.90-1.18) | 0.91 (0.79-1.04) | 1.02 (0.84-1.25) |
| *P* |  | 0.661 | 0.155 | 0.81 |
| *P*-heterogeneity, *I^2^* |  | 0.044, 75.2% | 0.235, 29.2% | 0.048, 74.4% |
| Sex |  |  |  |  |
| Male |  |  |  |  |
| Cases, N (%) | 1135 (30.63) | 1154 (31.15) | 1058 (28.56) | 358 (9.66) |
| Controls, N (%) | 1300 (31.15) | 1272 (30.47) | 1225 (29.35) | 377 (9.03) |
| OR (95% CI) | Reference | 1.08 (0.96-1.21) | 0.99 (0.88-1.12) | 1.08 (0.91-1.28) |
| *P* |  | 0.209 | 0.886 | 0.4 |
| Female |  |  |  |  |
| Cases, N (%) | 345 (28.12) | 409 (33.33) | 335 (27.30) | 138 (11.25) |
| Controls, N (%) | 606 (30.54) | 549 (27.67) | 659 (33.22) | 170 (8.57) |
| OR (95% CI) | Reference | 1.29 (1.07-1.56) | 0.90 (0.74-1.08) | 1.42 (1.09-1.85) |
| *P* |  | 7.31×10^-3^ | 0.252 | 9.60×10^-3^ |
| *P*-heterogeneity, *I^2^* |  | 0.109, 61.1% | 0.371, 0% | 0.086, 66.1% |
| Study site |  |  |  |  |
| Nanjing/Beijing |  |  |  |  |
| Cases, N (%) | 301 (29.92) | 373 (37.08) | 239 (23.76) | 93 (9.24) |
| Controls, N (%) | 676 (29.74) | 740 (32.56) | 643 (28.29) | 214 (9.41) |
| OR (95% CI) | Reference | 1.16 (0.96-1.40) | 0.87 (0.71-1.06) | 0.98 (0.74-1.30) |
| *P* |  | 0.123 | 0.174 | 0.900 |
| NCI |  |  |  |  |
| Cases, N (%) | 478 (29.41) | 457 (28.12) | 530 (32.62) | 160 (9.85) |
| Controls, N (%) | 696 (33.14) | 525 (25.00) | 697 (33.19) | 182 (6.87) |
| OR (95% CI) | Reference | 1.26 (1.06-1.50) | 1.13 (0.96-1.33) | 1.29 (1.01-1.65) |
| *P* |  | 8.19×10^-3^ | 0.156 | 0.040 |
| Jiangsu/Ningxia |  |  |  |  |
| Cases, N (%) | 701 (30.46) | 733 (31.86) | 624 (27.12) | 243 (10.56) |
| Controls, N (%) | 534 (29.91) | 556 (31.15) | 544 (30.48) | 151 (8.46) |
| OR (95% CI) | Reference | 1.01 (0.86-1.19) | 0.89 (0.75-1.05) | 1.21 (0.96-1.54) |
| *P* |  | 0.870 | 0.157 | 0.114 |
| *P*-heterogeneity, *I^2^* |  | 0.184, 41% | 0.066, 63.2% | 0.334, 8.8% |
| Tumor site |  |  |  |  |
| Cardia |  |  |  |  |
| Cases, N (%) | 424 (33.20) | 368 (28.82) | 354 (27.72) | 131 (10.26) |
| Controls, N (%) | 1906 (30.82) | 1821 (29.44) | 1884 (30.46) | 574 (9.28) |
| OR (95% CI) | Reference | 0.90 (0.77-1.05) | 0.84 (0.72-0.98) | 1.05 (0.84-1.31) |
| *P* |  | 0.169 | 0.027 | 0.639 |
| Noncardia |  |  |  |  |
| Cases, N (%) | 601 (29.09) | 733 (35.48) | 513 (24.83) | 219 (10.60) |
| Controls, N (%) | 1906 (30.82) | 1821 (29.44) | 1884 (30.46) | 574 (9.28) |
| OR (95% CI) | Reference | 1.28 (1.13-1.45) | 0.88 (0.77-1.01) | 1.29 (1.07-1.55) |
| *P* |  | 1.16×10^-4^ | 0.071 | 6.19×10^-3^ |
| *P*-heterogeneity, *I^2^* |  | 5.00×10^-4^, 91.8% | 0.617, 0% | 0.165, 48.2% |

CI, confidence interval; OR, odds ratio.
