# Supplementary material for: Blood groups A and AB are associated with increased gastric cancer risk: evidence from a large genetic study and systematic review
Source: BMC Cancer. 2019 Feb 21;19:164. doi: 10.1186/s12885-019-5355-4 (PMC6385454; doi:10.1186/s12885-019-5355-4)
Supplement: Supplementary file 3 — Figure S1. The flowchart of literature search and study inclusion. (Docx 26 KB) (DOCX 26 kb) [file 12885_2019_5355_MOESM3_ESM.docx]

Potentially relevant articles identified from literature search (n=324)

Articles excluded based on scanning titles and abstracts (n=204)

Candidate articles for further review (n=120)

Articles excluded based on full-text reading (n=93)

Data not available (n=75)

Articles not relevant to the study

(n=15)

Reviews or meta-analysis (n=3)

Articles identified in the reference list of the candidate articles (n=12)

Articles included in the meta-analysis (n=40)

Case-control study (n=33)

Nested case-control study (n=3)

Cohort study (n=4)

This study (n=1)

Additional file 3 Figure S1. The flowchart of literature search and study inclusion.
