# Supplementary material for: Blood groups A and AB are associated with increased gastric cancer risk: evidence from a large genetic study and systematic review
Source: BMC Cancer. 2019 Feb 21;19:164. doi: 10.1186/s12885-019-5355-4 (PMC6385454; doi:10.1186/s12885-019-5355-4)
Supplement: Supplementary file 4 — Table S3. Summaries of the studies included in the meta-analysis of ABO blood groups and gastric cancer risk. (DOCX 21 kb) [file 12885_2019_5355_MOESM4_ESM.docx]

| Additional file 4: Table S3. Summaries of the studies included in the meta-analysis of ABO blood groups and gastric cancer risk. | | | | | | | |
| --- | --- | --- | --- | --- | --- | --- | --- |
| Study | Publication year | Country | Ethnicity | Study design | Source of controls | Variables adjusted or matched | Quality score |
|  |  |  |  |  |  |  |  |
| Hollander et al. | 1953 | Switzerland | Caucasian | Case-control | NA | - | - |
| Aird et al. | 1953 | UK | Caucasian | Case-control | Hospital-based/Voluntary donors | - | 10 |
| Wallace et al. | 1954 | UK | Caucasian | Case-control | Voluntary donors | - | - |
| Koster et al. | 1955 | Denmark | Caucasian | Case-control | Patients' relatives/Voluntary donors/factory workers/soldiers | - | 7 |
| Billington et al. | 1956 | Australia | Caucasian | Case-control | Voluntary donors | - | 10 |
| Buckwalter et al. | 1957 | USA | Caucasian | Case-control | Voluntary donors | - | 7 |
| Segi et al. | 1957 | Japan | Asian | Case-control | Population-based | - | - |
| Eisenberg et al. | 1958 | USA | Caucasian | Case-control | Voluntary donors | - | 10 |
| Beasley et al. | 1960 | UK | Caucasian | Case-control | Population-based | - | 9 |
| Doll et al. | 1960 | UK | Caucasian | Case-control | Hospital-based | - | 7 |
| Doll et al. | 1961 | UK | Caucasian | Case-control | Hospital-based/Voluntary donors/members of the hospital staff/medical colleagues/personal friends/spouses of patients | - | 10 |
| Newman et al. | 1961 | USA | Caucasian | Case-control | Hospital-based/Voluntary donors | - | 9 |
| McConnell et al. | 1961 | NA | NA | Case-control | NA | - | - |
| Lisker et al. | 1964 | Mexico | Mixed | Case-control | Hospital-based | - | 9 |
| Hartmann et al. | 1964 | Norway | Caucasian | Case-control | Population-based | - | 6 |
| Hoskins et al. | 1965 | USA | Caucasian | Case-control | Voluntary donors | - | 8 |
| Hoskins et al. | 1965 | USA | Caucasian | Case-control | Hospital-based/ Voluntary donors | - | 8 |
| Glober et al. | 1966 | UK | Caucasian | Case-control | Voluntary donors | - | 7 |
| Ray et al. | 1980 | India | Asian | Nested case-control | Voluntary donors | Age, sex, race, site, and date of serum collection | 9 |
| Parsonnet et al. | 1997 | USA | Caucasian | Nested case-control | Population-based | - | 11 |
| Lissowska et al. | 1999 | Poland | Caucasian | Case-control | Population-based | - | 9 |
| Klaamas et al. | 1999 | Italy | Caucasian | Case-control | Voluntary donors | - | 9 |
| Su et al. | 2001 | China | Asian | Case-control | Voluntary donors | - | 9 |
| Zivanovic-Posilovic et al. | 2003 | Croatia | Caucasian | Case-control | Hospital-based | - | 8 |
| Sharara et al. | 2006 | Lebanon | Mixed | Case-control | Voluntary donors | - | 10 |
| El Hajj et al. | 2007 | Lebanon | Mixed | Case-control | Hospital-based/Voluntary donors | - | 8 |
| Iodice et al. | 2010 | Italy | Caucasian | Case-control | Hospital-based | - | 10 |
| Qiu et al. | 2010 | China | Asian | Case-control | Hospital-based | - | 10 |
| Edgren et al. | 2010 | North Europe | Caucasian | Cohort | Voluntary donors | Age, sex, country, and calendar period of observation | 11 |
| Nakao et al. | 2011 | Japan | Asian | Case-control | Hospital-based | Age, sex, smoking status, drinking habit, and family history of GC | 10 |
| Gong et al. | 2012 | China | Asian | Case-control | Hospital-based | - | 10 |
| Song et al. | 2012 | Korea | Asian | Case-control | Voluntary donors | Age, sex, smoking and drinking habits | 8 |
| Wang et al. | 2012 | China | Asian | Case-control | Voluntary donors | - | 7 |
| Li et al. | 2014 | China | Asian | Case-control | Hospital-based | Age and sex | 10 |
| Duell et al. | 2015 | Europe | Caucasian | Nested case-control | Population-based | Age, sex, and country | 9 |
| Sun et al. | 2015 | Taiwan | Asian | Cohort | Population-based | Age, sex, education level, smoking status, alcohol alcohol drinking, physical activity, and body mass index | 10 |
| Hsiao et al. | 2015 | Taiwan | Asian | Cohort | Population-based | Age, smoking status, alcohol drinking, hypertension, diabetes, and BMI | 11 |
| Emadi et al. | 2015 | Iran | Asian | Cohort | Population-based | Sex, ethnicity, place of residence, education, smoking, opium use, and socioeconomic status index | 10 |
| Sooyeon et al. | 2016 | Korea | Asian | Case-control | Hospital-based | - | 10 |
| This study | - | China | Asian | Case-control | Population-based/Hospital-based | Age, sex, and study site | 10 |
