# Supplementary material for: Blood groups A and AB are associated with increased gastric cancer risk: evidence from a large genetic study and systematic review
Source: BMC Cancer. 2019 Feb 21;19:164. doi: 10.1186/s12885-019-5355-4 (PMC6385454; doi:10.1186/s12885-019-5355-4)
Supplement: Supplementary file 5 — Figure S2. Begg’s funnel plots for ABO blood group and gastric cancer risk. Figs. A-C are funnel plots for blood group A (A), B (B), AB (C) verse group O. The vertical axis represents the log-transformed odds ratios (ORs). The horizontal axis represents the standard errors (SEs) of log-transformed ORs. The funnel plots are drawn with 95% confidence intervals. (DOCX 80 kb) [file 12885_2019_5355_MOESM5_ESM.docx]

**A**


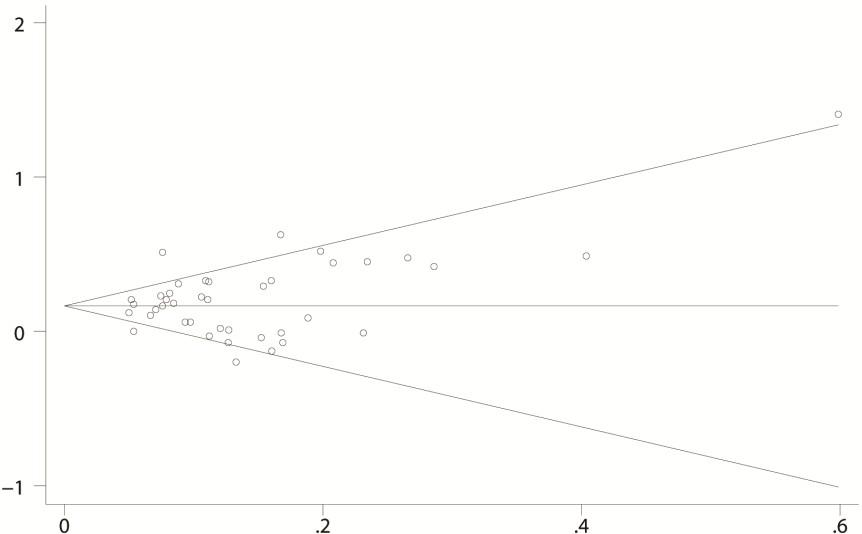


**Log-transformed OR**

**Standard error of log-transformed OR**

**B**


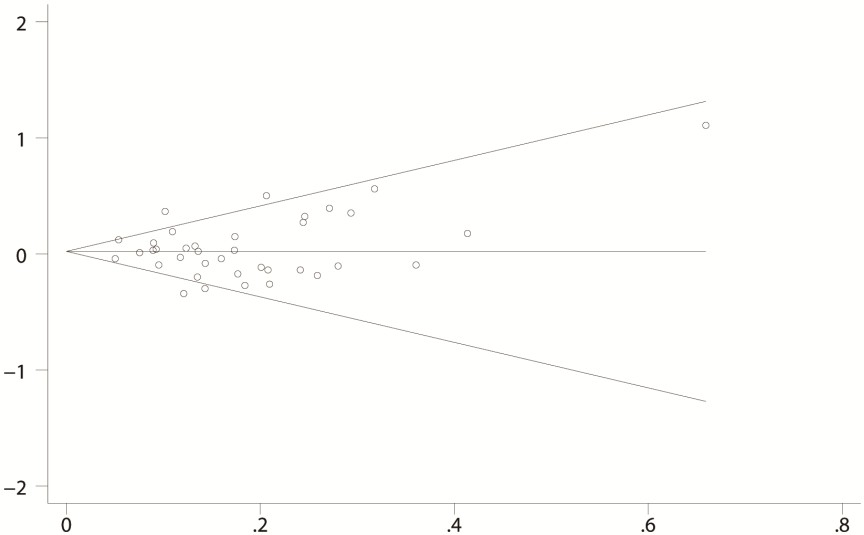


**Log-transformed OR**

**Standard error of log-transformed OR**

**C**


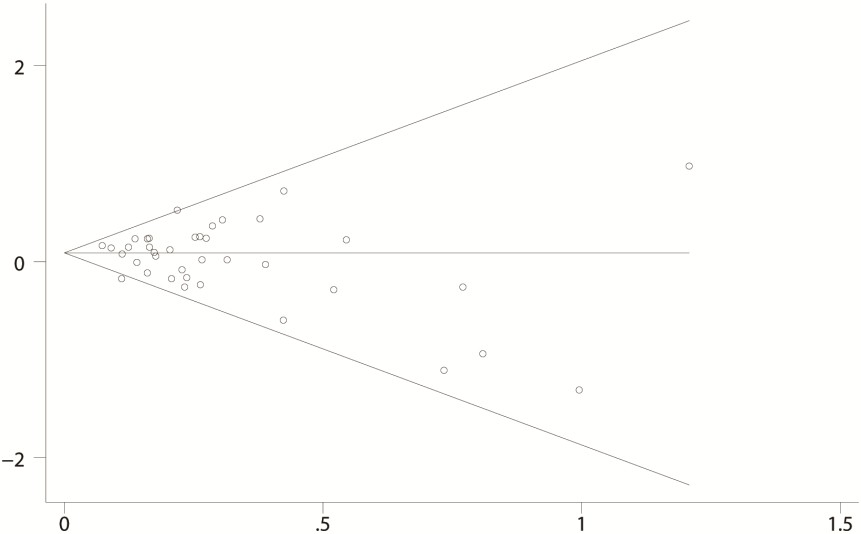


**Standard error of log-transformed OR**

**Log-transformed OR**

Additional file 5: Figure S2. Begg’s funnel plots for ABO blood group and gastric cancer risk. Figures A-C are funnel plots for blood group A (A), B (B), AB (C) verse group O. The vertical axis represents the log-transformed odds ratios (ORs). The horizontal axis represents the standard errors (SEs) of log-transformed ORs. The funnel plots are drawn with 95% confidence intervals.
