# Supplementary material for: Blood groups A and AB are associated with increased gastric cancer risk: evidence from a large genetic study and systematic review
Source: BMC Cancer. 2019 Feb 21;19:164. doi: 10.1186/s12885-019-5355-4 (PMC6385454; doi:10.1186/s12885-019-5355-4)
Supplement: Supplementary file 6 — Table S4. Subgroup analyses stratified by potential modifying factors. (DOCX 20 kb) [file 12885_2019_5355_MOESM6_ESM.docx]

Additional file 6: Table S4. Subgroup analyses stratified by potential modifying factors.

| Subgroups | Type A vs. O | | |  | Type B vs. O | | |  | Type AB vs. O | | |
| --- | --- | --- | --- | --- | --- | --- | --- | --- | --- | --- | --- |
|  | N^a^ | OR (95% CI) | *P*^b^ |  | N^a^ | OR (95% CI) | *P*^b^ |  | N^a^ | OR (95% CI) | *P*^b^ |
| All studies | 40 | 1.19 (1.13-1.25) | - |  | 38 | 1.02 (0.98-1.06) | - |  | 38 | 1.09 (1.03-1.16) | - |
| Ethnicity |  |  |  |  |  |  |  |  |  |  |  |
| Asians | 14 | 1.19(1.08-1.31) | 0.161 |  | 14 | 1.02(0.97-1.07) | 0.689 |  | 14 | 1.11(1.03-1.19) | 0.350 |
| Caucasians | 22 | 1.18(1.12-1.24) |  |  | 20 | 1.02(0.96-1.10) |  |  | 20 | 1.07(0.97-1.18) |  |
| Mixed | 3 | 1.72(1.17-2.55) |  |  | 3 | 1.20(0.85-1.70) |  |  | 3 | 1.32(0.68-2.57) |  |
| Publication year |  |  |  |  |  |  |  |  |  |  |  |
| Before 2000 | 22 | 1.18(1.13-1.24) | 0.692 |  | 21 | 1.07(1.00-1.14) | 0.041 |  | 21 | 0.99(0.90-1.09) | 0.012 |
| 2000 and after | 18 | 1.21(1.09-1.34) |  |  | 17 | 0.99(0.94-1.04) |  |  | 17 | 1.16(1.07-1.25) |  |
| Quality score |  |  |  |  |  |  |  |  |  |  |  |
| < 9 | 11 | 1.26(1.14-1.40) | 0.190 |  | 10 | 1.02(0.94-1.11) | 0.771 |  | 10 | 1.11 (0.99-1.24) | 0.004 |
| ≥ 9 | 25 | 1.16(1.09-1.24) |  |  | 24 | 1.03 (0.98-1.08) |  |  | 24 | 1.14 (1.06-1.22) |  |
| Sample size |  |  |  |  |  |  |  |  |  |  |  |
| <=7050 | 20 | 1.17(1.07-1.29) | 0.753 |  | 18 | 0.94(0.86-1.02) | 0.028 |  | 18 | 1.09(0.97-1.22) | 0.894 |
| >7050 | 20 | 1.20(1.12-1.27) |  |  | 20 | 1.05(1.00-1.09) |  |  | 20 | 1.10(1.03-1.16) |  |
| Source of control |  |  |  |  |  |  |  |  |  |  |  |
| Voluntary donors included | 19 | 1.22(1.13-1.32) | 0.272 |  | 19 | 1.09(1.03-1.15) | 0.003 |  | 19 | 1.13(1.04-1.23) | 0.208 |
| Without voluntary donors | 19 | 1.15(1.08-1.23) |  |  | 17 | 0.95(0.90-1.01) |  |  | 17 | 1.07(0.98-1.17) |  |
| Prevalence of *Helicobacter pylori* infection | |  |  |  |  |  |  |  |  |  |  |
| Low | 20 | 1.18(1.13-1.23) | 0.180 |  | 20 | 1.01(0.95-1.07) | 0.597 |  | 20 | 1.03(0.94-1.13) | 0.068 |
| High | 19 | 1.19(1.13-1.25) |  |  | 17 | 1.03(0.98-1.09) |  |  | 17 | 1.14(1.06-1.23) |  |

CI, confidence interval; OR, odds ratio. ^a^ The number of studies included. ^b^ *P* for heterogeneity test between strata.
